# Tyrosine Kinases in Autoimmune and Inflammatory Skin Diseases

**DOI:** 10.3389/fimmu.2019.01862

**Published:** 2019-08-09

**Authors:** Kata P. Szilveszter, Tamás Németh, Attila Mócsai

**Affiliations:** Department of Physiology, Semmelweis University School of Medicine, Budapest, Hungary

**Keywords:** tyrosine kinases, signaling, Syk, Jak, Src-family, dermatitis, psoriasis, autoimmune blistering diseases

## Abstract

Tyrosine kinases relay signals from diverse leukocyte antigen receptors, innate immune receptors, and cytokine receptors, and therefore mediate the recruitment and activation of various leukocyte populations. Non-receptor tyrosine kinases of the Jak, Src, Syk, and Btk families play major roles in various immune-mediated disorders, and small-molecule tyrosine kinase inhibitors are emerging novel therapeutics in a number of those diseases. Autoimmune and inflammatory skin diseases represent a broad spectrum of immune-mediated diseases. Genetic and pharmacological studies in humans and mice support the role of tyrosine kinases in several inflammatory skin diseases. Atopic dermatitis and psoriasis are characterized by an inflammatory microenvironment which activates cytokine receptors coupled to the Jak-Stat signaling pathway. Jak kinases are also implicated in alopecia areata and vitiligo, skin disorders mediated by cytotoxic T lymphocytes. Genetic studies indicate a critical role for Src-family kinases and Syk in animal models of autoantibody-mediated blistering skin diseases. Here, we review the various tyrosine kinase signaling pathways and their role in various autoimmune and inflammatory skin diseases. Special emphasis will be placed on identification of potential therapeutic targets, as well as on ongoing preclinical and clinical studies for the treatment of inflammatory skin diseases by small-molecule tyrosine kinase inhibitors.

## Introduction

Tyrosine kinases are intracellular enzymes mediating tyrosine phosphorylation of downstream molecules. They play a critical role in signal transduction by various cell surface receptors including, among others, growth factor receptors, adhesion receptors, immunoreceptors, and cytokine receptors. Given their role in multiple signaling processes and disease pathogenesis, tyrosine kinases have emerged as excellent therapeutic targets for the targeted therapy of various diseases. Indeed, small molecule tyrosine kinase inhibitors became important contributors to the pharmacological control of a diverse array of diseases including various malignant processes and immune-mediated diseases such as autoimmune and inflammatory conditions (1).

Inflammatory joint diseases such as rheumatoid arthritis have been in the focus of the development of tyrosine kinase inhibitors for therapeutic purposes within the area of immune mediated diseases (2). This has culminated in the regulatory approval of Jak inhibitors for the treatment of rheumatoid arthritis and certain related disease states (3). Besides inflammatory arthritis, inflammatory skin diseases are another major group of diseases with a major pathogenetic component of various immune cells and immunological pathways. Several lines of evidence indicate the contribution of various tyrosine kinases to the development and progression of diverse inflammatory skin diseases. Those issues suggest that tyrosine kinase inhibitors may provide therapeutic benefit in inflammatory skin diseases.

In this review article, we summarize our current knowledge and understanding of the role of tyrosine kinases in autoimmune and inflammatory skin diseases. We first provide an overview of the various receptor and non-receptor tyrosine kinases and their role in immunological and inflammatory processes. We then summarize the role of the various tyrosine kinases in specific autoimmune or inflammatory skin diseases. Special emphasis is placed on genetic studies in mice and humans indicating a role for tyrosine kinase pathways in inflammatory skin diseases, as well as the preclinical and clinical development of tyrosine kinases inhibitors for the targeted pharmacological therapy of those diseases.

## Signal Transduction by Tyrosine Kinases

### Non-receptor Tyrosine Kinases

Non-receptor tyrosine kinases are intracellular tyrosine kinases without a direct role in sensing extracellular cues. Nevertheless, these tyrosine kinases are often coupled to various cell surface receptors and are intimately involved in the transmission of extracellular signals to downstream intracellular signaling pathways and cellular effector functions.

There are a total of 10 different non-receptor tyrosine kinase families. Of those, we will discuss Janus kinases (also known as Jak-family kinases), Src-family kinases, and the Syk tyrosine kinase, as well as members of the Btk kinase family.

#### Janus Kinase Family

The Janus kinase family consists of four members: Jak1, Jak2, Jak3, and Tyk2. While Jak1, Jak2, and Tyk2 are ubiquitously expressed, the expression of Jak3 is limited to the hematopoietic compartment.

**Figure 1:**
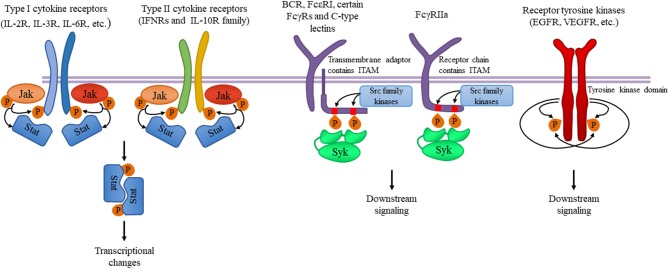
Tyrosine kinases and their signaling pathways. Type I and type II cytokine receptors utilize Janus kinases for the initiation of downstream signaling. Type I cytokine receptor superfamily shares a common amino acid motif WSXWS. Within this family receptors containing the common γ chain (γc) recognize IL-2, IL-4, and IL-13 among others utilizing Jak1 and Jak3. Cytokines including IL-3 and IL-6 are also recognized by type I cytokine receptors acting through Jak1/Jak2 heterodimers or Jak2 homodimers, respectively. The type II cytokine receptor family includes receptors activated by interferons (IFNs) and the IL-10 family utilizing heterodimers of Jak1 along with Jak2 or Tyk2. Ligand binding leads to Jak activation resulting in phosphorylation of the receptor and downstream signal transducers and activators known as Stats mediating transcriptional changes (more information in the text). Src-family kinases and Syk are involved in several immune cell signaling pathways like immunoreceptor, integrin and C-type lectin signaling. Upon ligand binding, activation of Src-family kinases leads to the phosphorylation of tyrosine residues in immunoreceptor tyrosine-based activation motifs (ITAMs), that can be part of a transmembrane adaptor molecule like in case of B cell receptor (BCR), FcεRI, and certain FcγRs and C-type lectins, or of the receptor chain itself like in FcγRIIa in humans. Syk is recruited to the dually phosphorylated ITAMs and becomes activated resulting in the recruitment and activation of various further adapter proteins promoting downstream signaling. Receptor tyrosine kinases, for example EGFR and VEGFR have intrinsic tyrosine kinase activity leading to auto-and transphosphorylation of the receptor chains upon ligand binding. Recruitment of several adaptors and effector molecules through SH2 and phosphotyrosine binding domains mediate downstream signaling.

These kinases are primarily involved in the signal transduction of various cytokine receptors which are grouped into type I (extracellular WSXWS sequence present; e.g., IL-2, IL-6, GH, EPO, G-CSF, and GM-CSF receptors) and type II (no extracellular WSXWS sequence; e.g., IFN-α, IFN-β, and IL-10 receptors) cytokine receptors. Since Jak-coupled cytokine receptors act as dimers, Jak family kinase activity is also mediated by involving two Jak-family kinases. In most cases, the two cooperating kinases are different (“heterodimers,” although they do not form a firm dimer), although Jak2 can also cooperate with another Jak2 molecule (“homodimer”). Upon ligand binding, the conformational changes of the receptor and/or ligand-induced dimerization promotes Jak activation, which leads to autophosphorylation of tyrosine residues of the kinase itself, further augmenting its kinase activity. Jaks then phosphorylate the receptor chains, allowing the recruitment of various signal transducer and activator of transcription (Stat) transcription factors. Stat molecules are then also phosphorylated by Jak kinases, leading to dimerization and translocation to the nucleus where they activate or repress gene expression and influence epigenetic alterations (Figure 1). These basic signaling principles are conserved across the diverse array of different biological functions of the Jak-Stat signaling pathway.

Despite the complex and promiscuous nature of receptor association of the different Jak family kinases, human inherited traits and mouse genetic studies have revealed several critical functions of the different Jak kinases. Jak1 is essential for the signaling through type II cytokine receptors (such as IFN receptors), as well as through receptors that utilize the common γ-chain (γc) or the shared gp130 subunit. Jak1 deficiency in mice leads to defective lymphoid development and neurological defects resulting in perinatal lethality without disturbing other hemopoietic lineages (4). Signaling downstream of type II IFNs and receptors with shared gp130 subunit also require Jak2 (besides Jak1), whereas, Jak2 mediated signaling is not required for lymphoid development. IL-3 receptor and several hormone-like receptors (EPO, TPO, GH, PRL) signal through Jak2 alone. Deletion of Jak2 leads to embryonic lethality due to failure of definitive erythropoiesis in mice, likely due to the role of Jak2 in signaling by cytokine receptors involved in the regulation of hematopoiesis and, especially, erythropoiesis (5, 6). Jak3 expression is essentially limited to hematopoietic cells and it is known that it constitutively and exclusively binds to γc-containing receptors including IL-2 and IL-4 receptors. Jak3 mutation in humans leads to severe combined immunodeficiency (7, 8) and studies using *Jak3*^−/−^ mice further confirmed the critical role of Jak3 in lymphoid development (9, 10). Tyk2 is important in IL-12 and IL-23-mediated T cell responses and IFN signaling (11, 12). *Tyk2*^−/−^ mice are viable but susceptible to infections, and macrophages fail to respond to LPS both *in vitro* and *in vivo* (13, 14).

Given their central role in cytokine signaling it is not surprising that Jaks have a role in several immune mediated diseases involving autoimmunity, transplant rejection, and malignancies. Therefore, pharmacological targeting of Jaks was plausible and Jak inhibitors have been extensively studied in several clinical studies. A critical aspect of Jak inhibitors is their selectivity profile for the different Jak family kinases which determines the spectrum of their biological effects. Table 1 provides a list and the selectivity profile of currently available Jak inhibitors based on cell-free assays. The mechanism of action of those drugs is competitive binding to the ATP binding site of the kinase domain therefore inhibiting phosphorylation and activation of Jaks, except for the case of PF6615600 and BMS986165 (15). PF6615600 mediates a covalent, irreversible Jak3 inhibition through a non-conserved Cys residue in the ATP binding pocket, whereas BMS986165 binds to the pseudokinase domain of Tyk2 (15). First generation Jak inhibitors (tofacitinib, ruxolitinib, baricitinib, and oclacitinib) tend to be less selective among the Jak family kinases due to structural similarities in the ATP binding site of different Jaks, whereas more selective inhibitors were developed during later stages of drug development. Discrepancies between biochemical and cellular potencies of Jak inhibitors have been reported, potentially due to the dominant role of one Jak over another in certain cytokine signaling pathways (16).

**Table 1:** Jak inhibitors and their selectivity profile.

**Compound**	**Primary target(s)**	**IC**_****50****_ **in cell free assay (nM)**			
		**Jak1**	**Jak2**	**Jak3**	**Tyk2**
Tofacitinib	Jak3, Jak2, and Jak1	112	20	1	34
Ruxolitinib	Jak1 and Jak2	3.3	2.8	428	19
Baricitinib	Jak1 and Jak2	5.9	5.7	560	53
Delgocitinib	Jak1, Jak2	2.8	2.6	13	58
Momelotinib	Jak1, Jak2	11	18	155	n.a
Filgotinib	Jak1 > Jak2	10	28	810	116
Solcitinib	Jak1	8–9	108	539	225
Upadacitinib	Jak1	47	120	2,300	4,700
Itacitinib	Jak1	2	63	>2,000	795
Abrocitinib	Jak1	29	803	>10,000	1,253
PF-06651600	Jak3	>10,000	>10,000	33,1	>10,000
PF-06700841	Tyk2 > Jak1	n.a	n.a	n.a	n.a
BMS986165	Tyk2	n.a	n.a	n.a	2–14
SAR-20347	Tyk2	23	26	41	0.6

*Obtained from MedChemExpress and Selleckchem. n.a, not available*.

The most studied Jak inhibitor is tofacitinib, which ameliorated autoimmune arthritides in various animal models (17–20) and proved to be effective in several phase II and III studies in the treatment of rheumatoid arthritis, leading to regulatory approval by both the FDA and EMA (2, 21). Jak inhibitors are also currently under investigation in other immune mediated diseases like inflammatory bowel disease, transplant rejection, and multiple dermatological disorders. Excellent reviews about the current state of Jak inhibitors and ongoing clinical trials have been published recently (3, 15, 22).

#### Src-Family Kinases and the Syk Tyrosine Kinase

The Src kinase family includes nine members (including Hck, Fgr, Lyn, and Lck) which are involved in many signaling pathways in immune cells including immunoreceptor as well as integrin signaling. Src-family kinase activity is regulated by tyrosine phosphorylation and Src homology 2 and 3 (SH2 and SH3) mediated protein-protein interactions with partner proteins containing phosphotyrosine or proline-rich motifs, respectively. Src-family kinases are ubiquitously expressed, although different cells express different family members. Within the immune system, T cells express Lck and Fyn, B cells express Fyn, Lyn, and Blk, and myeloid cells express Hck, Fgr, and Lyn (23). Spleen tyrosine kinase (Syk) is a tandem SH2 domain-containing enzyme acting mostly downstream of Src-family kinases. Syk is expressed in most hematopoietic lineage cells except for T-cells (and, partially, NK-cells) where a closely related kinase, ZAP-70 is expressed and performs a similar function (24).

Immunoreceptors such as B cell receptors (BCR), T cell receptors (TCR), and various activating Fc receptors of innate immune cells are physically associated with transmembrane adapter proteins carrying immunoreceptor tyrosine-based activation motifs (ITAMs). Ligand-receptor interaction results in the enzymatic activation of Src-family kinases phosphorylating the ITAM motifs in the receptor subunits (24). Dually phosphorylated ITAMs are recognized by the tandem SH2 domains of Syk (or ZAP-70 in T cells and NK cells), leading to the recruitment and activation of various further adapter proteins and the activation of several downstream signaling pathways leading to cellular responses (Figure 1). Leukocyte integrin “outside-in” signaling also requires Src family kinases and Syk, resulting in adhesion-induced activation of immune cells (24–28). Some inhibitory receptors containing immunoreceptor tyrosine-based inhibitory motifs (ITIMs) also act through Src-family kinases (mostly Lyn), resulting in phosphatase activation and downmodulating of activating signals.

T cell development requires TCR-based signaling and Src-family kinases, particularly Lck. In case of B lymphocytes, Lyn kinase has primary role in BCR signaling. Paradoxically, B cell-specific deletion of Lyn not only results in the expected defects in B cell development, but also leads to autoimmunity (29). Myeloid cells primarily express Hck, Fgr, and Lyn which have a critical but overlapping role in the activation of neutrophils and macrophages through Fcγ receptors, as well as through β_1_ and β_2_ integrins. A prominent feature of Src-family kinases is a significant functional overlap between individual family members. Therefore, in contrast to Jak kinases, individual Src-family kinases are not essential for a given response and complete inhibition of a signaling pathway often requires combinational deletion of multiple kinases in myeloid cells. FcγR mediated phagocytosis is slowed in macrophages lacking Hck, Fgr, and Lyn (30). Adhesion-induced activation is also abrogated in neutrophils lacking Hck, Fgr, and Lyn including oxidative burst, degranulation, and cell spreading (28). Their role has also been shown in chemokine and cytokine responses (31). Moreover, *Hck*^−/−^*Lyn*^−/−^*Fgr*^−/−^ triple knockout but not single or double knockout animals were completely protected from autoantibody induced arthritis due to the defective generation of inflammatory environment without affecting the intrinsic migratory capacity of myeloid cells (32).

Currently available Src-family inhibitors have limited selectivity, also inhibiting various other tyrosine kinases such as c-Kit, EGFR, or Abl (summarized in Table 2). Those inhibitors are often used in cancer therapy based on their effects on kinases other than Src-family kinases. As an example, dasatinib and bosutinib are potent multi-target inhibitors of Abl, Kit, and several members of the Src kinase family. Beside their therapeutic use in hematological malignancies, they have been found to be relevant in inflammatory conditions as well both *in vitro* and *in vivo* in immune-mediated experimental models (48–51).

**Table 2:** Inhibitors of the Src-family and Syk.

**Compound**	**Primary target(s)** **(IC_**50**_ in cell free assay)**	**Other targets** **(IC_**50**_ in cell free assay)**	**Clinical relevance**
Dasatinib	Src (0.8 nM), Abl (<1 nM)	c-Kit (79 nM)	Approved in chronic myeloid leukemia and acute lymphoblastic leukemia (33)
Bosutinib (34)	Src (1.2 nM), Abl (1 nM)	n.a	Approved in chronic myeloid leukemia (35)
PP1 (36)	Lck (5 nM), Fyn (6 nM)	Hck (20 nM), Src (170 nM), Bcr-Abl (1 μM), Kit (75 nM), EGFR (250 nM)	–
PP2 (36)	Lck (4 nM), Fyn (5 nM), Hck (5 nM)	EGFR (480 nM), other 56 kinases at 10 μM (37)	–
Fostamatinib (38)	Syk (41 nM), Flt3	79 kinases <100 nM (39)	Approved in immune thrombocytopenia (40)
Entospletinib (41)	Syk (7.7 nM)	TNK1 (<100 nM) (39)	Investigated in hematological malignancies (42)
P505-15 (43)	Syk (1–2 nM)	Fgr (81 nM), Yes (123 nM), MLK1 (88 nM)	–
RO9021 (44)	Syk (5.6 nM)	n.a	–
PRT318 (45)	Syk (4 nM)	n.a	–
TAK-659 (46)	Syk (3.2 nM)	Flt3 (4.6 nM), ZAP-70 (75 nM), Jak3 (114 nM), VEGFR2 (135 nM)	Investigated in hematological malignancies and solid tumors (47)

*Obtained from MedChemExpress and Selleckchem. n.a, not available*.

Syk and ZAP-70 are also essential for the development of mature B and T cells, respectively (24, 52, 53). Syk deficiency leads to perinatal lethality due to defective separation of lymphoid and blood vessels (54). In the myeloid compartment, Syk is a key protein mediating Fc receptor and integrin mediated signaling and also mediates downstream signaling of C-type lectins like Dectin-1 recognizing fungal antigens (55, 56). Deficiency of the Syk kinase produces profound defects in neutrophil/macrophage integrin signaling and responses to immune complexes, resulting in significantly reduced stimulation of respiratory burst, degranulation and cell spreading (57–59). Syk-deficient bone marrow chimeras proved to be completely protected from autoantibody-induced arthritis that is due to enzymes specifically expressed in neutrophils (60–62). The partially selective Syk inhibitor fostamatinib showed clinical benefit in rheumatoid arthritis patients (63) and has been also investigated in other autoimmune and allergic diseases but considerable adverse events possibly due to its poor selectivity profile led to the suspension of further investigations in RA. New and more specific Syk inhibitors have been developed in the past few years (summarized in Table 2) that show promising results in this regard according to *in vitro* results, animal models of autoimmune arthritis and phase I clinical trials (41, 43, 44, 64).

Cerdulatinib and gusacitinib represent dual inhibitors of Syk and Jak kinases and cerdulatinib demonstrated efficacy in experimental arthritis (65). The concept that dual inhibition may result in a stronger therapeutic response is favorable, however it can also represent a limitation by the increased risk of toxicity.

#### Bruton's Tyrosine Kinase

Bruton's tyrosine kinase (Btk) is involved in the development and activation of B cells through BCR and Toll-like receptor (TLR) signaling (66). Patients with loss-of-function mutations in the Btk gene suffer from immunodeficiency due to the absence of mature B cells and immunoglobulins (67, 68). Similarly, deficiency of Btk in mice results in an impaired differentiation of B cells (69). In addition, transgenic mice that overexpress human Btk display systemic autoimmune response with spontaneous germinal center formation, increased cytokine production (IFNγ and IL-6) and anti-nuclear autoantibodies (ANAs) (70). Btk and other members of the Btk family like Tec kinase are also expressed in myeloid cells regulating maturation and effector function (71). Btk inhibitors interacting with the ATP binding site have been developed and proved to be effective in several systemic autoimmune mouse models like arthritis and lupus models (72, 73).

### Receptor Tyrosine Kinases

Receptor tyrosine kinases represent a large family of receptors recognizing various hormones, cytokines, and growth factors (74). They form dimeric combinations upon ligand binding resulting in auto- and transphosphorylation and the recruitment and activation of effectors containing SH2 and phosphotyrosine binding domains, leading to multiple downstream signaling (Figure 1).

EGFR and its related receptors, PDGFRs, VEGF receptors and their intact signaling are essential for normal embryonic development and adult tissue homeostasis including cell survival, proliferation, adhesion and migration. Their deregulation has been associated with many human diseases, including immune-mediated disorders and cancer. Targeted therapy by receptor tyrosine kinase inhibitors revolutionized cancer therapy (75). VEGF receptors mediate angiogenesis and lymphangiogenesis during the inflammation process regulating immune cell recruitment and resolution of inflammation.

## Tyrosine Kinases in Inflammatory Skin Diseases

### Atopic Dermatitis

Atopic dermatitis (AD) is the most common inflammatory skin disease. A T_H_2 dominated immune response is essential in the pathogenesis causing eczematous dermatitis with intense pruritus accompanied by elevated serum concentrations of IgE. AD is commonly associated with other T_H_2 mediated allergic diseases called the “atopic-allergic march” (76).

#### Pathogenesis—Pivotal Role of Barrier Disruption and Subsequent TSLP Production

The fundamental lesion is currently thought to be an impaired barrier function that can be due to disrupted expression of essential barrier proteins like filaggrin (77, 78). Subsequent increased penetration of cutaneous and environmental antigens leads to the production of keratinocyte-derived cytokines including thymic stromal lymphopoietin (TSLP). TSLP is thought to be a critical factor driving the pathogenesis of atopic diseases. TSLP receptor is widely expressed in cells that contribute to AD (dendritic cells, T cells, B cells, mast cells, eosinophils, epithelial cells, and sensory neurons) utilizing the Jak-Stat pathway in humans. However, interestingly, it seems that murine TSLP receptor activates Stats by the Btk-family kinase Tec without the involvement of Janus kinases (79). Tamoxifen-induced keratinocyte-specific TSLP-deficient mice displayed drastically reduced allergic skin inflammation in a tape-stripping- and ovalbumin-induced AD model accompanied by the impairment of T_H_2 response and allergen-induced sensitization (80). In contrast, overexpression of TSLP in keratinocytes triggered massive itching behavior together with the development of AD-like dermatitis (81). Therefore, intradermal TSLP injection is often used to induce AD-like dermatitis in mice. Wilson et al. showed that it triggers itch sensation within minutes independently from the presence of adaptive immunity or mast cells (82). They also showed that TSLP receptors are present in sensory neurons innervating the skin and TSLP-evoked neuronal activation was responsible for itch sensation.

#### T_H_2 Mediated Allergic Responses—Key Role of T_H_2 Cytokines in AD

The importance of the T_H_2 pathway is further supported by the observation that transgenic mice overexpressing the T_H_2 cytokines IL-4 or IL-13 spontaneously develop skin inflammation that is frequently used as an animal model for AD (83). IL-4 and IL-13 are recognized by the type I cytokine receptor (IL-4 receptor) containing the γc subunit that signals through Jak1 and Jak3. Besides regulating IgE production in B cells and promoting the differentiation of T_H_2 lymphocytes, the IL4 receptor is also constitutively expressed by keratinocytes. Stimulation of IL-4 receptors leads to cytokine and chemokine production and downregulation of genes involved in keratinocyte differentiation (such as filaggrin) *in vitro*. This suggests that IL-4 receptor signaling in keratinocytes can further contribute to barrier impairment and inflammation in AD (84–87). Moreover, type 2 cytokines are also capable of activating sensory neurons directly depending on IL-4 receptor and Jak signaling, thus contributing to the development of chronic itch in AD (88). Dupilumab, a monoclonal antibody against the α subunit of the IL-4 receptors that blocks signaling from both IL-4 and IL-13 was effective in phase III studies (89, 90) and it was the first biologic agent approved by the FDA and EMA in the treatment of adults with moderate-to-severe atopic dermatitis (91).

#### Contribution of IgE-Mediated Signaling to the Allergic Response in AD

Elevated IgE levels and IgE autoreactivity were also suggested to contribute to the development and severity of AD. Beside mast cells and basophils, significant FcεRI expression has been shown in professional antigen presenting cells in atopic skin lesions. FcεRIs crosslinked with IgE are expected to use ITAM-dependent pathways including Src-family kinases and Syk to facilitate degranulation, internalization of allergens and antigen presentation promoting T_H_2 immunity (92).

#### Chronic Phase—Transition Into T_H_1-Type Inflammation

T_H_1 and IFNγ-mediated responses are thought to dominate the chronic phase of the disease (83). Animal models of hapten-, or allergen-induced contact dermatitis resemble pathogenetic features of both acute and chronic AD involving the disruption of the barrier as a primary event followed by sensitization, inflammation, increased epidermal proliferation, and changes in keratinocyte-differentiation (83).

#### The Jak-Stat Pathway in Preclinical Studies

**Figure 2:**
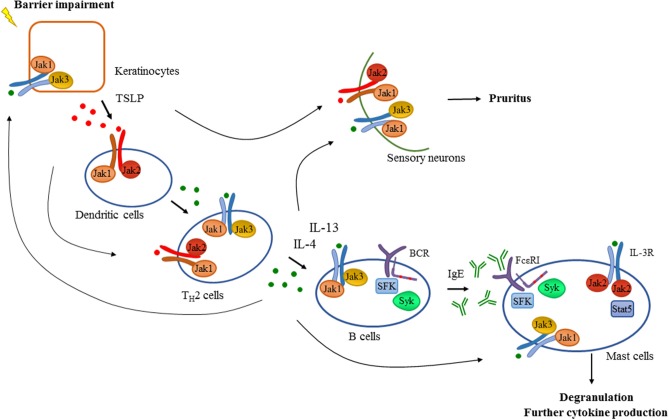
Tyrosine kinases in the development of atopic dermatitis. Barrier impairment is one of the initial steps in the pathogenesis of atopic dermatitis which leads to the production of TSLP by keratinocytes that activates dendritic cells in a Jak1/Jak2 dependent manner in the skin. Proinflammatory mediators produced by dendritic cells and keratinocytes drive the activation and differentiation of CD4^+^ T cells toward the T_H_2 phenotype. Activated T_H_2 cells produce several cytokines including IL-4 and IL-13 which promote isotype switch and IgE production in B cells acting through receptors with the common γ chain (γc) utilizing the Jak1/Jak3 heterodimer. Additionally, intact B cell receptor (BCR) signaling involving Src-family kinases (SFK) and Syk is essential for normal B cell development. Downstream effector cells like mast cells recognize IgE through FcεRI in a Src-family and Syk dependent manner leading to degranulation and the production of proinflammatory mediators contributing to the development of atopic dermatitis. There are several feedback loops and shortcuts in this pathway facilitating inflammation utilizing tyrosine kinases. T_H_2 cytokines also promote the activation of keratinocytes and effector immune cells like mast cells requiring intact Jak signaling. Activation of mast cells through IL-3R/Jak2/Stat5 pathway was also found to be important in allergen-induced dermatitis in mice. Furthermore, TSLP and T_H_2 cytokines acting through a Jak dependent manner in sensory neurons were found to mediate pruritus in atopic dermatitis in addition to mast cell-derived compounds such as histamine.

The above mentioned pathogenetic features strongly suggest the role of the Jak-Stat pathway in AD (Figure 2). Accordingly, Yasuda et al. showed that gain of function mutation in Jak1 resulted in a spontaneous dermatitis phenotype (93). Generation of bone marrow chimeras revealed that Jak1 expression in non-hematopoietic cells was responsible for the development of dermatitis, but Jak1 acting in immune cells exacerbated the dermatitis symptoms and disease severity. They claimed that a possible molecular mechanism behind these findings was that hyperactivation of Jak1 pathway in epidermal keratinocytes resulted in a skin barrier defect due to the overexpression of serine proteases (93). The Jak3 inhibitor tofacitinib reduced ear-swelling and scratching behavior in an allergen-induced dermatitis model, especially upon topical application (94). Inhibition of Jak1 and Jak2 by topically applied ruxolitinib or momelotinib successfully decreased inflammation in a hapten-induced hypersensitivity model as well as in TSLP-induced dermatitis in mice together with the down-regulation of mRNA expression of IL-4, IL-5, IFNγ, and TSLP in the skin (95, 96). Oral administration of delgocitinib (JTE-052) efficiently reduced inflammation in a murine model of hapten-induced hypersensitivity. Moreover, delgocitinib inhibited proliferation and activation of T cells, but did not affect the number of DCs migrated to the draining lymph node during the sensitization phase (97). Delgocitinib was even found to be superior to conventional therapeutic agents like cyclosporine or tacrolimus ointment in hapten- and in TSLP-induced murine dermatitis models with respect to ear thickness, microscopic phenotype, inflammatory cytokine production, and serum IgE level (98). Another study suggested that delgocitinib might directly enhance keratinocyte differentiation *in vitro* (87). Delgocitinib treatment increased the expression of filaggrin and loricrin, genes that are known to be involved in keratinocyte differentiation, in primary human keratinocytes. In addition, IL-4 receptor-mediated downregulation of these genes was reversed upon delgocitinib treatment. Transepidermal water loss was also reduced upon delgocitinib treatment *in vivo* in a murine dry skin model which does not induce immune cell infiltration, indicating that Jak inhibition can improve skin barrier function independently of affecting immune cell activation (87). Taken together, the Jak-Stat pathway seems to be a central component of AD development, mediating multiple aspects of the pathogenesis such as type II cytokine signaling and TSLP-mediated inflammation, itching, keratinocyte disruption, and barrier impairment together with IFNγ-driven responses in the chronic phase. These findings strongly suggest a therapeutic utility of Jak inhibitors in human AD.

#### Jak Inhibitors in Clinical Trials

Given the substantial interest toward Jak inhibitors, a large amount of data is available from case reports, retrospective studies and open-label studies in the field of treatment of inflammatory skin diseases. In this review we are focusing on randomized, placebo-controlled phase II-III clinical trials that meet the standards of accepted evidence-based medicine (Table 3).

**Table 3:** Phase II and III clinical trials studying Jak inhibitors in atopic dermatitis.

**Drug name**	**Identifier**	**Status**	**Phase, administration**	**Enrollment**	**Duration**
Tofacitinib	NCT02001181	Completed (99)	IIa, topical	69	4-week
Ruxolitinib	NCT03011892	Completed (100)	IIb, topical	307	8-week dose-ranging and additional 4-week optional open-label treatment
	NCT03745638; NCT03745651	Underway (101)	III, topical	1,200	8-week and long-term safety extension period
Delgocitinib	NCT03725722	Recruiting	IIb, topical	250	8-week dose-ranging
Baricitinib	NCT02576938	Completed (102)	II, per os	124	16-week in combination with TCS
	NCT03334396; NCT03334422	Met primary endpoint (103)	III, per os	1,350	16-week
	NCT03334435	Recruiting	III, per os	1,500	52-week to evaluate long-term safety
	NCT03428100	Recruiting	III, per os	500	16-week in those who cannot have cyclosporin
	NCT03435081	Recruiting	III, per os	450	16-week dose-ranging study
	NCT03559270	Recruiting	III, per os	300	2-year in those who have completed NCT03435081
	NCT03733301	Recruiting	III, per os	300	16-week in combination with TCS
Abrocitinib	NCT02780167	Completed, results online	IIb, per os	269	12-week dose-range study
	NCT03796676	Recruiting	III, per os	225, adolescents	12-week with other topical therapy
	NCT03575871; NCT03349060	Recruiting	III, per os	375	12-week
	NCT03422822	Recruiting	III, per os	2,300	Approximately 2 years who have completed a qualifying phase III study
	NCT03627767	Recruiting	III, per os	1,370	Over 40 weeks in those who responded well to an initial 12-week treatment
	NCT03720470	Recruiting	III, per os	700	12-week, efficacy compared to dupilumab at 2 weeks
Upadacitinib	NCT02925117	Completed (104)	III, per os	166	16-week
	NCT03738397	Recruiting	III, per os	650	24-week treatment, 12-week follow-up
	NCT03661138	Recruiting	III, per os	264	Up to 141 weeks, evaluating safety
	NCT03568318	Recruiting	III, per os	810	16-week combined with TCS
	NCT03607422; NCT03569293	Recruiting	III, per os	810	16-week

Both topical tofacitinib and ruxolitinib treatment significantly improved skin inflammation and pruritus in AD patients in phase II studies (99, 100). In addition, the TRuE-AD phase III clinical trial has just started with the aim of assessing the efficacy of topical ruxolitinib treatment with long-term safety extension period in a larger cohort (101). Studies evaluating efficacy and safety of novel, more selective Jak inhibitors have also shown promising results. Patients treated with baricitinib in combination with topical corticosteroids achieved greater reduction in disease severity than corticosteroids alone (102). In another phase III trial, baricitinib as a monotherapy met its primary endpoint in AD patients (103). Per os abrocitinib and upadacitinib also significantly improved the Eczema Area and Severity Index and reduced pruritus compared to placebo [NCT02780167, (104), respectively]. Emerging number of studies are currently recruiting patients to evaluate efficacy and long-term safety up to 2 years in AD patients (Table 3).

### Psoriasis

#### IL-23 Is Crucial to Activate IL-17-Mediated Effector Responses

**Figure 3:**
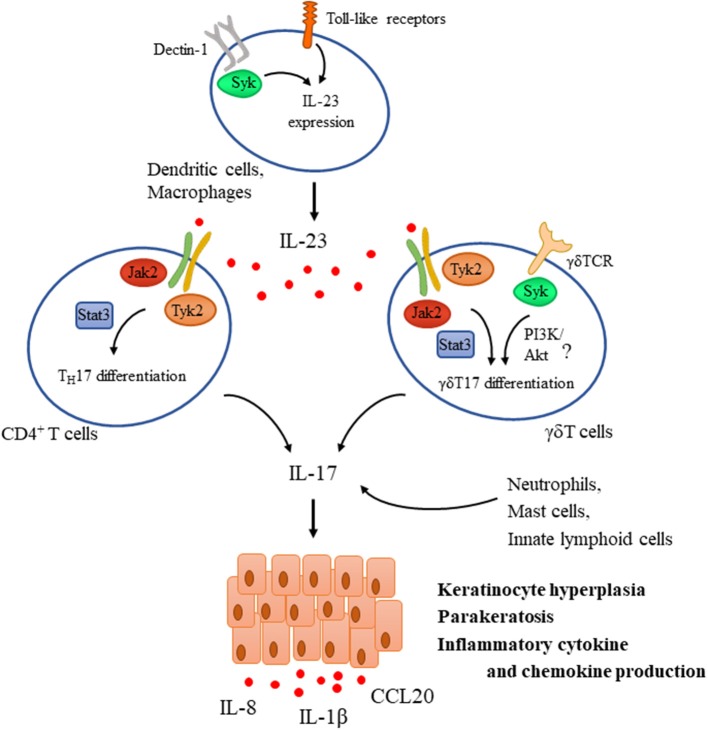
Tyrosine kinases in the IL-23/IL-17 axis during psoriasis pathogenesis. IL-23 is a key mediator driving psoriasis pathogenesis. It is expressed by dendritic cells upon their activation through Toll-like receptors (TLR) and Dectin-1, the latter one utilizing Syk. IL-23 promotes the differentiation of CD4^+^ T cells and γδT cells as well toward T_H_17 and γδT17, respectively via a Jak2/Tyk2-Stat3-mediated pathway and also induces the expression of IL-17. TCR activation in γδ T cells was also proposed to be important for mediating IL-17 production and psoriatic skin lesions, utilizing Syk and probably the PI3K/Akt pathway. In addition to T_H_17 and γδT17 cells, other sources of IL-17 include neutrophils, mast cells, and innate lymphoid cells. IL-17 then acts as an effector acting mainly on keratinocytes mediating hyperproliferation, parakeratosis, and production of several inflammatory chemokines and cytokines like IL-1β, IL-8, and CCL20.

Psoriasis is a common chronic inflammatory skin disease characterized by epidermal hyperplasia and parakeratosis together with the accumulation of inflammatory cells in the dermis, clinically causing red scaly papules and plaques. Activation of the IL-23/IL-17 axis plays a pivotal role in the pathogenesis of the disease (Figure 3). Resident immune cells such as dendritic cells and macrophages are considered to be initially activated through Toll like receptors that lead to the production of cytokines including IL-23 and TNFα that are known to be critical for IL-17 production in human psoriatic skin (105, 106). In fact, intradermal injection of IL-23 alone results in a skin pathology that strongly mimics human disease and is therefore commonly used as an animal model of psoriasis (107). IL-23 is known to induce the differentiation of T_H_17 cells that are considered as a major source of IL-17 in humans (106). However, activated dermal γδ T cells were also implicated as major IL-17-producing cells upon IL-23 stimulation in mouse models of psoriasis (108). IL-17 and IL-22 (another T_H_17 cytokine) then act as effectors inducing keratinocyte proliferation and hyperkeratosis. They also enhance the production of inflammatory cytokines and chemokines (including IL-1β, CCL20, and IL-8) by keratinocytes, leading to recruitment of other effector cells like neutrophils, further contributing to tissue damage and establishing the inflammatory milieu (109).

#### Jak-Stat Signaling in Psoriasis

A number of novel biological therapies (including monoclonal antibodies against IL-23, IL-17, and IL-17R) have been approved for the treatment of psoriasis in the last few years (110). IL-23 receptors rely on Jak2/Tyk2 heterodimer-mediated signaling, implicating their role in the pathogenesis of the disease. Several genes of Jak-Stat signaling pathway have also shown to be associated with psoriasis (111). This was further supported by a genetic approach showing that Tyk2-deficient mice had significantly reduced ear swelling and epidermal hyperplasia upon injection with IL-23. In addition, infiltration of various leukocytes (including different T cell subsets, neutrophils, and macrophages) and the production of the pro-inflammatory cytokines IL-17 and IL-22 were also impaired in the absence of Tyk2 (112). Downstream signaling components like Stat3, a key factor in T_H_17 differentiation, was also found to be upregulated in human psoriatic lesions (113). Sano et al. showed that constitutive expression of Stat3 in keratinocytes resulted in a dermatitis phenotype closely resembling psoriasis (113). Epidermal hyperplasia, parakeratosis and dermal infiltration of immune cells occurred upon tape stripping or wounding of the skin, and in some mice, it occurred even spontaneously. The development of psoriatic lesions in these mice required both hyperactive Stat3 in keratinocytes and activated T cells in the dermis. Furthermore, inhibition of Stat3 with decoy oligonucleotides successfully inhibited disease development and even reversed disease severity showing that Stat3 may be an important regulator of genes in keratinocytes in the development of psoriasis cooperating with T cells (113).

#### Jak Inhibitors in the Treatment of Psoriasis

Pharmacological inhibition of the Jak-Stat pathway showed promising results in murine models of psoriasis reducing disease pathology, keratinocyte-activation, and proinflammatory cytokine levels (95, 98, 114). In addition, a number of clinical trials investigate the effect of different Jak inhibitors in psoriasis (Table 4). Short-term oral tofacitinib therapy resulted in significant clinical improvement in patients with moderate-to-severe plaque psoriasis (116) along with reducing epidermal thickness, DC and T cell numbers and the expression of psoriasis-related genes in the lesional skin (115). Several additional phase III studies were completed evaluating the long-term safety and efficacy of the treatment. Oral administration of tofacitinib was non-inferior to parenteral etanercept indicating that tofacitinib may provide a more convenient therapeutic option (117). Tofacitinib also demonstrated sustained efficacy in patients with psoriasis through up to 52 months and was well-tolerated with an acceptable safety profile detailed later (118, 121, 122, 130). In a 56-week withdrawal and retreatment study, patients who received continuous treatment maintained a response more effectively, however, 60% of patients who relapsed upon tofacitinib withdrawal recaptured a response with tofacitinib (119).

**Table 4:** Phase II and III clinical trials studying Jak inhibitors in psoriasis.

**Drug name**	**Identifier**	**Status**	**Phase, administration**	**Enrollment**	**Duration**
Tofacitinib	NCT01710046	Completed (115)	IIa, per os	12	12-week
	NCT00678210	Completed (116)	IIb, per os	197	12-week
	NCT01241591	Completed (117)	III, per os	1,101	12-week non-inferiority trial compared to etanercept
	NCT01519089	Completed (118)	III, per os	95	52-week evaluating long-term safety
	NCT01186744	Completed (119)	III, per os	666	56-week withdrawal and retreatment study
	NCT01276639 and NCT01309737	Completed (120)	III, per os	901 and 960	52-week
	NCT01815424	Completed (121)	III, per os	266	52-week
	NCT01163253	Terminated as it met its objectives (122)	III, per os	2,867	median duration 35.6 months open-label extension study who completed qualifying phase II/III studies
	NCT01831466	Completed (123)	IIb, topical	430	12-week
Ruxolitinib	NCT00820950	Completed (124)	II, topical	29	28-day
	NCT00778700	Completed, no results available	II, topical	199	12-week
Baricitinib	NCT01490632	Completed (125)	IIb, per os	271	12-week
Abrocitinib	NCT02201524	Terminated (126)	II, per os	59	4-week terminated due to changes in sponsors development priorities
PF-06700841	NCT02969018	Completed	IIa, per os	212	12-week, results online
	NCT03850483	Not yet recruiting	IIb, topical	240	12-week
Itacitinib	NCT01634087	Completed (127)	II, per os	50	28-day
BMS986165	NCT02931838	Completed (128)	II, per os	267	12-week
	NCT03624127 and NCT03611751	Recruiting	III, per os	600 and 1,000	Non-inferiority study compared to apremilast
Solcitinib	NCT01782664	Completed (129)	IIa, per os	68	12-week

Topical treatment provides an excellent opportunity to overcome possible systemic adverse effects. Tofacitinib ointment also showed greater efficacy compared to vehicle at week 8, but failed to be superior to placebo at week 12 in a phase IIb study (123). Topical ruxolitinib was found to be well-tolerated, safe, and efficacious in short-term treatment in a smaller cohort of patients (124).

Oral treatment of novel, more selective inhibitors also improved symptoms and were well-tolerated in patients with psoriasis in phase II trials (125–127, 129). Since IL-12/IL-23-mediated signaling relies on Jak2/Tyk2 heterodimers, specific inhibition of these kinases may provide further improvement in psoriasis patients. Due to the critical role of Jak2 in hemopoietic development, inhibition of Tyk2 seem to be plausible in the treatment of psoriatic patients. Indeed, BMS986165 showed promising results in a 12-week phase II trial enrolling 267 patients (128) and a phase III study is currently recruiting patients to compare oral BMS986165 treatment to placebo and currently available treatment apremilast (NCT03624127 and NCT03611751).

#### Syk Tyrosine Kinase in IL-17-Mediated Inflammation

Little is known about the role of Syk in psoriasis. The pattern recognition receptor Dectin-1 was implicated in the disease process, suggesting that recognition of fungal antigens in a Syk and CARD9 dependent manner promotes the maturation of DCs and their ability to induce IL-17 production by T_H_17 cells (55). In addition, another source of IL-17 is γδ T cells, which utilize Syk as a dominant proximal kinase of the γδ TCR signaling pathway. Furthermore, skin inflammation was ameliorated in mice lacking the adaptor molecule RhoH that recruits Syk to the TCR in imiquimod-induced psoriasis model (131). These findings suggest that Syk may contribute to IL-17 production, but its actual relevance in case of psoriasis needs further investigation.

#### Role of Growth Factor Receptors in AD and Psoriasis

EGF receptor family members in keratinocytes facilitate epidermal differentiation and plays a crucial role in wound healing as well as in carcinogenesis. Though epidermal hyperplasia is a hallmark of both AD and psoriasis, the contribution of EGF receptor signaling to inflammatory skin disorders is poorly understood. Psoriatic lesions are known to overexpress EGFR and ligands like amphiregulin. Transgenic overexpression of amphiregulin in either basal or suprabasal epidermis causes severe psoriasis-like hyperplasia and skin inflammation in mice (132, 133). In line with that, neutralizing antibodies against amphiregulin reduce epidermal thickness of human psoriatic lesions transplanted onto mice with severe combined immunodeficiency (134). However, mice lacking another EGFR ligand epiregulin develop severe chronic dermatitis showing complicated modulating role of EGFR signaling pathways in the epidermis (135). Mice lacking epidermal EGFR spontaneously develop skin inflammation, decreased host defense and deficient skin barrier function (136). Proinflammatory cytokines such as IFNγ was also suggested to transactivate EGFR leading to the downregulation of the expression of chemokines like CCL2, CCL5, and CXCL10. In line with that, EGFR inhibition resulted in the aggravation of allergic contact dermatitis in mice by enhanced chemokine production of keratinocytes, promoting subsequent leukocyte recruitment (137). In contrast, IL-8 gene expression is actively induced by the EGFR ligands in keratinocytes (135, 137). In turn, IL-8 could contribute to activating the metalloprotease-dependent release of EGFR ligands by acting on its specific receptor in cancer cells (138), indicating the possibility of a positive feedback loop both for epidermal hyperplasia and neutrophil accumulation.

It is also well-known that EGFR inhibitor therapy in malignancies often causes inflammatory or toxic effects on the skin, and such side effects even act as strong predictors of good response to treatment (139).

Psoriasis and atopic dermatitis are both characterized by altered angiogenesis and lymphangiogenesis (140). Hyperplastic hyperpermeable dermal blood vessels can be detected in psoriatic skin lesions and transgenic delivery of VEGF to the skin results in a profound inflammatory skin condition resembling psoriasis (141) while topical application of VEGFR inhibitor successfully prevented disease development in the mouse model of psoriasis (142). Interestingly, stimulation of lymphangiogenesis by VEGFR-3 or via administration of its ligand VEGF-C inhibited inflammatory cell infiltration by oxazolone-induced skin inflammation (143, 144). This indicates that blood vessels contribute to the development of inflammatory environment by helping inflammatory cell infiltration while lymphatic vessels may limit skin inflammation by helping their elimination. Thus, selectivity can be especially important upon targeting VEGFRs in malignancies.

### Alopecia Areata and Vitiligo

Both alopecia areata and vitiligo are characterized by IFNγ producing autoreactive cytotoxic T lymphocytes that attack hair follicles and melanocytes, respectively.

Alopecia areata (AA) is the main cause of non-scarring hair loss most commonly occurring in the scalp. The upregulation of several Jak-Stat pathway components downstream of γ-chain containing cytokines (which are known to promote the activity and survival of IFNγ-producing cytotoxic T cells) was detected in AA skin both from humans and mice. In a mouse model of AA, systemic administration of Jak inhibitors successfully prevented the development of disease. Moreover, both systemic and topical administration was able to reverse established disease and even promoted hair regrowth (145). Gene expression and immunofluorescent studies from mouse skin showed that expression and activity of the Jak-Stat pathway is dynamically changing during the hair follicle cycle. Moreover, Jak inhibition promoted the entry into the hair cycle and subsequent hair regrowth by activating hair follicle stem cells in healthy mice and also in lymphocyte-deficient mouse strains (146). This dual effect further justifies the investigation of Jak inhibitors in the treatment of AA. Some open label phase II clinical studies showed that Jak inhibitor treatment resulted in significant hair regrowth and improvement of AA in patients with better response if administered orally instead of topical formulations. Hair loss typically reoccurred after discontinuation of therapy within months (147–149). There are several active randomized, double-blind, placebo-controlled phase II clinical trials on the efficacy and safety of topical and oral Jak inhibitors in AA (Table 5), promising further insight into these issues in the near future.

**Table 5:** Phase II and III clinical trials studying Jak inhibitors in alopecia areata and vitiligo.

**Disease**	**Drug name**	**Identifier**	**Status**	**Phase, administration**	**Enrollment**	**Duration**
Alopecia areata	Baricitinib	NCT03570749	Ongoing	II/III, per os	725	36-week
	PF-06651600 and PF-06700841	NCT02974868	Ongoing	IIa, per os	142	24-week with extension period up to 2 years
	Delgocitinib	NCT02561585	Results submitted	II, topical	31	12-week
Vitiligo	Ruxolitinib	NCT03099304	Ongoing	II, topical	157	1-year with 1 year open-label extension
	PF-06651600 and PF06700841	NCT03715829	Recruiting	IIb, per os	330	60-week with 24 weeks dose ranging and 24 week extension period

In case of vitiligo cytotoxic T cell-mediated melanocyte destruction causes depigmentation leading to the occurrence of white spots throughout the body surface of patients. IFNγ produced by activated melanocyte-specific cytotoxic T lymphocytes is strongly implicated in the disease pathogenesis by promoting further T cell accumulation in the skin through IFNγ-dependent chemokines like CXCL10 (150). In addition, IFNγ was found to directly induce melanocyte senescence and apoptosis of primary human melanocytes which could be attenuated by siRNA against Jak2 and Stat1, but not Jak1. IFNγ treatment also resulted in the accumulation of reactive oxygen species and the production of proinflammatory cytokines like IL-6 which are considered as important contributing factors facilitating a vitiligo-prone environment in the skin (151).

Given the multiple role of IFNγ in vitiligo, inhibition of the Jak-Stat pathway may represent a promising therapeutic strategy. Beside some case reports and retrospective studies showing benefits of Jak inhibitors treating vitiligo patients (152, 153), so far only one open label phase II proof of concept pilot trial has been completed. This showed that topical ruxolitinib treatment provided significant repigmentation in facial vitiligo in a small cohort of patients (154). A randomized double-blind, dose-ranging, placebo-controlled phase II trial is now ongoing for evaluating the efficacy of ruxolitinib cream in vitiligo (NCT03099304). Another randomized controlled phase IIb trial is currently recruiting patients for evaluating per os treatment with novel selective Jak inhibitors (NCT03715829, also see in Table 5).

An additional important aspect of AA and vitiligo is that these diseases mostly cause cosmetic concern with emotional problems. Therefore, analyzing the safety and benefit profile of potential treatments is extremely important and Jak inhibitors should be very carefully tested in this regard.

### Pemphigus

Pemphigus vulgaris and foliaceus are severe autoimmune blistering skin diseases with autoantibodies that are directed against the desmosomal cadherins, mainly desmoglein 3 (Dsg3) and Dsg1. These are required for the proper intercellular adhesion of keratinocytes and autoantibody deposition results in flaccid blister formation within the epidermis affecting mucous membranes and skin. Pemphigus vulgaris (PV) can be life threatening causing significant loss of the physical barrier. Application of artificial barriers like ointments can improve barrier function but there is an unmet need for definitive treatment.

Given the central role of Btk and Syk in B cell development and activation, these kinases may promote pathogenic antibody production in pemphigus. An inhibitor of Btk is under current evaluation in an open-label phase II clinical trial in pemphigus (NCT02704429) moreover, a randomized, double-blind placebo-controlled phase III study is already recruiting patients (NCT03762265).

IgG4 is the major subclass of autoantibodies in pemphigus and it is known to have limited ability to activate complement. Accordingly, acantholysis in pemphigus has been demonstrated to be independent from complement and Fc receptors (155, 156). Anti-Dsg3 IgG was able to directly induce the destruction of desmosomes by steric hindrance, promoting the internalization of Dsg3 (157, 158) and interfering with desmosome turnover (159). Recently it has been shown that Dsg3 transcription is negatively regulated by Stat3 in keratinocytes and corticosteroid treatment upregulates Dsg3 expression by inhibiting Stat3 through a yet unknown mechanism possibly involving Jaks (160). Those results suggest that specific inhibition of the Jak-Stat3 pathway may also be beneficial without the known adverse effects of steroids. T_H_2 cytokines like IL-4, IL-9, and IL-21, that utilize Jak-family kinases are known to contribute to the induction and regulation of autoantibody production in pemphigus (161, 162) therefore Jak inhibitors also serve as a potential treatment, but preclinical or clinical studies are yet to be done (163).

Binding of pathogenic IgG can also trigger outside-in signaling in keratinocytes eliciting acantholysis and apoptosis. Moreover, autoantibody binding also promoted secretion of inflammatory cytokines from keratinocytes, which may augment the pathogenic autoimmune response (164). However, the precise molecular mechanism is at present unclear. Several downstream mediators were implicated involving activation of EGFR-mediated signaling and focal adhesion kinase in keratinocytes (165–169). In a recent report, Src family kinases were implicated in autoantibody-mediated desmosome disassembly (170). Src phosphorylation was induced in keratinocytes upon *in vitro* antibody treatment obtained from PV patients. Loss of cell cohesion caused by anti-Dsg3 antibody was abolished upon Src inhibition by PP2 both *in vitro* and *in vivo* in a neonatal mouse model. However, inhibition of Src was not protective in some cases against PV-Ig-induced loss-of-keratinocyte-cohesion in keratinocyte monolayer, nor in intact human skin (170). These results underlie the need for further investigations of keratinocyte signaling in pemphigus pathogenesis.

### Pemphigoid Diseases

Pemphigoid diseases are characterized by autoantibody production against distinct components of the dermal-epidermal junction leading to dermal-epidermal separation and tense blister formation (171).

Bullous pemphigoid (BP) is the most prevalent autoimmune bullous disease. Antibody formation directed against key hemidesmosomal components BP180 (also called type XVII collagen), and/or BP230 results in subepidermal blistering phenotype. Urticarial plaques and pruritus are also present in most of the cases which is a unique symptom among the pemphigoid group. Epidermolysis bullosa acquisita (EBA) is a very rare disease characterized by autoantibodies directed against type VII collagen (C7), the key anchoring fibril in the upper dermis, also causing subepidermal blister formation (172).

#### Essential Role of Antibody-Deposition in Pemphigoid Diseases

**Figure 4:**
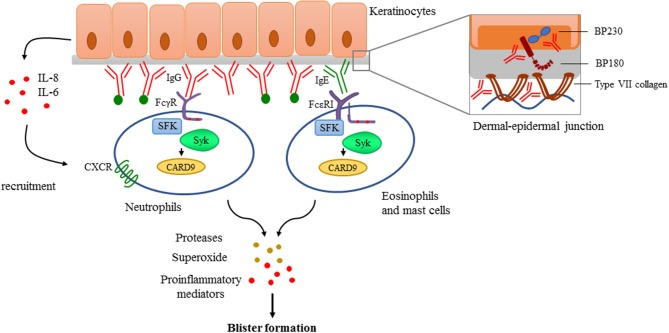
Antibody-mediated signaling in pemphigoid diseases. Pemphigoid diseases are characterized by autoantibody production against components of the dermal-epidermal junction (DEJ) like BP180 and BP230 in case of bullous pemphigoid, or against type VII collagen in patients with epidermolysis bullosa acquisita (Structure of the DEJ with special attention to the above-mentioned autoantigens is shown on the upper right panel). Autoantibody deposition along the DEJ results in the activation of the complement system (indicated with green dots) and initiation of keratinocyte-responses involving IL-6 and IL-8 production. This leads to recruitment of innate effector cells like neutrophils, eosinophils, and mast cells. Recognition of deposited immune complexes through Fc receptors utilizing Src-family kinases (SFK), Syk, and CARD9 is critical for the development of pemphigoid diseases, as shown by genetic studies using transgenic animals (see more detail in the text). Immune complex-mediated activation of effectors leads to the release of reactive oxygen species, proteases, and the production of proinflammatory mediators culminating into blister formation.

Antibody deposition to the basement membrane is the key feature in the pathogenesis, demonstrated by the fact that passive transfer of either anti-BP180 or anti-C7 antibodies isolated either from human patients or generated against human or murine antigens result in a severe blistering phenotype in mice (173–176). These approaches became useful experimental models to investigate the effector phase of autoantibody-mediated pemphigoid diseases. Several studies showed that deposition of antibodies are followed by the activation of the complement system and the recruitment of neutrophils mediating tissue damage and blister formation (177–181). Eosinophils and mast cells are also considered as contributors of blister formation by producing mediators facilitating further neutrophil recruitment and tissue damage (182–184). However, there are controversies in the literature especially regarding the role of mast cells (185). It has been proposed that autoantibodies can also directly affect epidermal cell—extracellular matrix integrity (186–188) and can trigger morphological and functional changes in keratinocytes (189) including IL-6 and IL-8 production (190) which can promote the recruitment of neutrophils (Figure 4).

#### Pathways Mediating Immune-Complex Recognition and Leukocyte Migration

Signaling through complement receptors and recognition of deposited immune complexes by neutrophils through activating Fcγ receptors was essential for blister formation both *in vitro* using human cryosections and in *in vivo* mouse models (191–193). FcεRI signaling in mast cells, eosinophils and basophils have also been implicated in immune complex recognition because IgE isotypes of autoantibodies can also be detected in many patients in addition to IgG (194, 195). Actual pathogenetic relevance of IgE and FcεRI was further supported by the fact that omalizumab treatment was able to reduce the number of blisters and itching in BP patients (196). However, the role of other potential participants, namely β_2_ integrins, which are known to be involved in the migration of neutrophils to the inflamed area, is not entirely clear. Mac-1-deficient neonatal mice developed impaired neutrophil infiltration and were resistant to blister formation after 24 h of a single anti-BP180 treatment (197). In contrast, another study in the experimental model of EBA induced by repeated anti-C7 injections found that the absence of Mac-1 led to an even exacerbated disease phenotype (198). Therefore, further experiments are needed to reveal the role of β_2_ integrins in pemphigoid diseases.

#### Src-Family Kinases and Syk in Pemphigoid Diseases

Fc receptor-mediated signaling is strongly dependent of intracellular tyrosine kinases like Src-family kinases and Syk (24, 25). Given the central role of immune complex recognition by Fc receptors of resident and recruited innate immune cells, Src-family kinases and Syk may have an important role in the development of pemphigoid diseases. Indeed, triple knockout mice lacking Hck, Fgr, and Lyn, three Src-family kinases expressed in the myeloid compartment, were completely protected in an autoantibody induced model of EBA (32). Neutrophils lacking Hck, Fgr, and Lyn also failed to produce superoxide in response to C7-containing immune complexes (199), which has been shown to be important in mediating tissue damage in the skin (200). The Syk tyrosine kinase, which is recruited to ITAM sequences phosphorylated by Src-family kinases, was also found indispensable for the effector phase of the disease (199, 201). Analysis of Syk-deficient bone marrow chimeras revealed that Syk deficiency completely protected mice from anti-C7 antibody-induced skin disease and abrogated the accumulation of key cytokines and chemokines, as well as the infiltration of leukocytes, at the site of inflammation (199). Moreover, Syk deficient neutrophils failed to release CXCL2 or leukotriene B_4_ upon activation by immobilized C7-anti-C7 immune complexes *in vitro*. Integrin signaling also acts through Src kinases and Syk, however, *in vivo* migratory capacity either of Syk deficient or Src-family triple knockout neutrophils remained unaffected (199). Furthermore, neutrophil-specific expression of the CARD9 adaptor protein was also found to contribute to the development of the disease, as complete or neutrophil-specific CARD9 deletion partially protected mice from anti-C7 antibody-induced skin inflammation, likely due to CARD9-dependent regulation of neutrophil gene expression changes (202).

Taken together, Fc receptors and β_2_ integrins signal through Src-family kinases and Syk which are indispensable for immune-complex and adhesion-induced activation of effector cells (neutrophils, macrophages and, possibly, eosinophils and mast cells) without affecting their intrinsic migratory capacity. Src and Syk kinases are also responsible for the amplification of the inflammation process through the release of mediators that recruit neutrophils and/or directly damage dermal-epidermal junction in experimental pemphigoid models like proteases and superoxide. Therefore, these non-receptor tyrosine kinases may be good candidates for therapeutic intervention in the future, even though the development of specific inhibitors has yet to be solved.

#### Presence of Jaks in Pemphigoid Diseases

Several proinflammatory cytokine levels are elevated in blister fluid of BP patients such as IL-1β, IL-4, IL-6, IL-8, and TSLP (172, 203–205), and many of them act through the Jak-Stat pathway. In line with that, the expression of Jak-Stat proteins was found to be also elevated in skin lesions of BP patients (206). A meeting abstract discussed that pharmacological inhibition of Jak2 impaired the induction of EBA by antibody transfer and had therapeutic effects too in immunization-induced EBA model (207). However, there are no clinical studies using Jak inhibitors in pemphigoid diseases. A case report has been published about successfully treating a BP patient with anti-IL-4 antibody (208). The low number of clinical studies in general with novel therapeutic options is possibly due to the fact that BP usually affects elderly patients, therefore one should very carefully balance risk-benefit ratio in case of novel systemic treatments with special attention to inflammation and carcinogenesis.

### Systemic Lupus Erythematosus

Almost all patients with systemic lupus erythematosus (SLE) develop lupus-specific cutaneous symptoms at some point in the disease course. There are also patients with cutaneous lupus that do not meet other diagnostic criteria for SLE. SLE is characterized by autoreactive B cells and autoantibody formation which proposes the role for Syk and Btk, non-receptor tyrosine kinases mediating BCR signaling, in disease development. The partially selective Syk inhibitor fostamatinib prevented the development of skin disease and significantly reduced established skin disease in lupus-prone mice (209). In addition, Btk inhibiton significantly attenuated the lupus-associated cutaneous disease phenotypes in mice (210). Systemic inflammatory conditions mediated by secretion of proinflammatory cytokines that act through Jaks is also important in SLE cutaneous manifestations (211–215). Indeed, Jak inhibition by ruxolitinib prevented the development of cutaneous lupus lesions in lupus-prone mice (216). However, baricitinib failed to improve skin manifestations in systemic lupus patients in a phase II trial, despite the fact that the overall systemic symptoms were effectively reduced by the drug (217).

### Neutrophilic Dermatoses

Neutrophilic dermatoses represent a group of disorders characterized by massive neutrophil infiltration in the skin without evidence of infection. Signs of systemic inflammation often accompanies skin pathology and they are often associated with malignancies and autoimmune diseases like RA and inflammatory bowel diseases. Interestingly, autoinflammatory diseases share various common features with neutrophilic dermatoses suggesting some similarities in the pathogenesis (218). Since an excellent review has been currently published about mechanisms of inflammation in neutrophil dermatoses (219), here we are focusing on evidence about the importance of tyrosine kinases.

Abnormalities in neutrophil function is obviously implicated in the pathogenesis. The SH2 domain-containing tyrosine phosphatase SHP-1 is essential for inhibiting proinflammatory signal transduction and loss-of-function mutation of SHP-1 in mice causes severe cutaneous inflammation resembling human neutrophilic dermatoses. Kanneganti and her group showed that IL-1α signaling through IL-1R and adaptor protein Myd88 drives inflammation in this model where tyrosine phosphorylation of Myd88 is counterregulated by SHP-1 and Syk (220). Dysfunction of SHP-1 leads to the release of Syk from inhibition resulting in excessive expression of proinflammatory mediators and other effector molecules. Downstream of Syk, the CARD9 adaptor protein was also found to be a key mediator in cutaneous inflammation in the aforementioned model (221).

Cytokine dysregulation are considered as contributing factors to the development of the disease. Elevation of serum G-CSF was detected in patients with active disease of unknown origin (222), moreover, G-CSF is a common cause of drug-induced neutrophilic dermatosis (223). G-CSFR signals through Jak2 suggesting a possible role of Jak2 creating cytokine dysregulation. In addition, increased expression of IFNγ was also described in patients and rare genetic autoinflammatory diseases characterized by high IFNγ production also represent neutrophilic dermatosis where Jak inhibitor therapy significantly improved symptoms in some cases (224).

## Adverse Events During JAK Inhibitor Treatment in Inflammatory Diseases

Safety information from long-term studies of Jak inhibitor treatment are limited due to the novelty of these drugs. Most data are available from RA patients (225), but there is slowly emerging information from patients with skin inflammation and several long-term safety-assessing trials are currently recruiting patients (Tables 3–5).

Safety profile is considered generally acceptable, infections and laboratory abnormalities can be observed as major adverse events (226). The increased risk of infections was similar to that observed by the use of biologics, with the exception of higher risk for varicella zoster infection which should be taken into consideration. Cytopenias, mostly anemia and neutropenia relatively often occur likely due to Jak2 inhibition but rarely severe. Elevated serum cholesterol level has been also mentioned in patients treated with Jak inhibitors (227). It was usually sustained after the first few months and was controllable by statin therapy. Among less frequent adverse events thromboembolism was documented, however most patients already suffer from increased risk due to chronic systemic inflammation which further complicates the picture (228). One of the most serious concerns is the possibility of the development of malignancies upon long-term Jak inhibitor treatment. Current studies have not shown higher risk (225), but longer follow-up is needed to properly address this matter.

Taken together, Jak inhibitor treatment has not higher risk than biologics overall. However, skin diseases often present as less severe but chronic symptoms where risk/benefit ratio should be carefully considered upon choosing appropriate treatment. Nevertheless, the possibility of topical application represents an excellent opportunity which deserve further elaboration.

## Conclusion and Future Directions

The highly effective treatment of various malignancies by tyrosine kinase inhibitors and the regulatory approval of Jak inhibitors for the targeted therapy of rheumatoid arthritis has generated major interest in the therapeutic targeting of other diseases, including inflammatory skin diseases, by tyrosine kinase inhibitors. Besides the most extensively studied Jak inhibitors, compounds targeting other kinases such as Syk, Src-family kinases, or Btk are also expected to emerge as new therapeutics for inflammatory dermatitis. The more and more detailed understanding of individual kinase family members and the development of novel inhibitors with more specifically tailored specificities toward individual kinases are expected to lead to more refined therapies driving the field toward personalized targeted therapeutic approaches. Skin diseases also provide unique opportunities for the development of novel small molecule therapeutics, mostly through the opportunity of topical application without systemic side effects. On the other hand, the cosmetic aspects of skin diseases and the role of skin and mucous membranes as critical barriers between the internal and external environment also present substantial challenges during the development of novel therapeutics. Taken together, the scientific community may expect exciting major advances in the field of understanding and targeting tyrosine kinases in inflammatory skin diseases in the coming years.

## Author Contributions

KS and AM wrote the manuscript. TN provided intellectual advice.

### Conflict of Interest Statement

The authors declare that the research was conducted in the absence of any commercial or financial relationships that could be construed as a potential conflict of interest.
